# 2-(3-Chloro­benzo­yl)-3-(3,4-di­chloro­phen­yl)-1-(4-ferrocenylphen­yl)guanidine

**DOI:** 10.1107/S1600536813021892

**Published:** 2013-08-10

**Authors:** Rukhsana Gul, Azim Khan, Amin Badshah, M. Nawaz Tahir

**Affiliations:** a Department of Chemistry, Gomal University, Dera Ismail Khan, K.P.K, Pakistan; b Department of Chemistry, Quaid-i-Azam University, Islamabad, Pakistan; c University of Sargodha, Department of Physics, Sargodha, Pakistan

## Abstract

In the title compound, [Fe(C_5_H_5_)(C_25_H_17_Cl_3_N_3_O)], the isolated cyclo­penta­dienyl (Cp) ring is disordered over two set of sites in a 0.577 (8):0.423 (8) ratio. The dihedral angle between the other Cp ring and its attached benzene ring is 13.6 (3)°, and that between the benzene ring and the guanidine group is 64.8 (2)°. One of the N—H groups forms both an intra- and an inter­molecular N—H⋯O hydrogen bond; the other N—H group does not form any hydrogen bonds. In the crystal, pairs of the inter­molecular N—H⋯O hydrogen bonds link the mol­ecules into inversion dimers.

## Related literature

For a related structure, see: Bequeath *et al.* (2007 ▶). For further synthetic details, see: Gul *et al.* (2013 ▶).
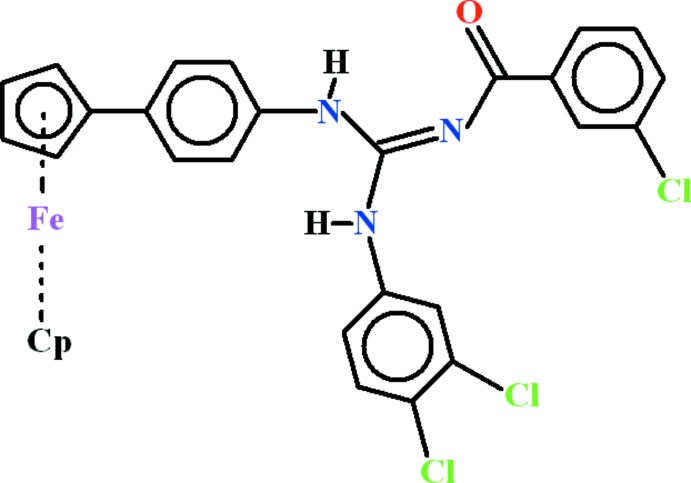


## Experimental

### 

#### Crystal data

[Fe(C_5_H_5_)(C_25_H_17_Cl_3_N_3_O)]
*M* *_r_* = 602.71
Monoclinic, 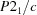

*a* = 17.674 (3) Å
*b* = 6.1352 (12) Å
*c* = 23.961 (5) Å
β = 95.359 (9)°
*V* = 2586.8 (9) Å^3^
*Z* = 4
Mo *K*α radiation
μ = 0.92 mm^−1^
*T* = 296 K
0.32 × 0.15 × 0.14 mm

#### Data collection

Bruker Kappa APEXII CCD diffractometer
Absorption correction: multi-scan (*SADABS*; Bruker, 2009) ▶ *T*_min_ = 0.757, *T*_max_ = 0.882
20020 measured reflections
4828 independent reflections
2612 reflections with *I* > 2σ(*I*)
*R*_int_ = 0.088

#### Refinement

*R*[*F* ^2^ > 2σ(*F* ^2^)] = 0.060
*wR*(*F* ^2^) = 0.122
*S* = 1.01
4828 reflections
319 parameters
H-atom parameters constrained
Δρ_max_ = 0.45 e Å^−3^
Δρ_min_ = −0.36 e Å^−3^

### 

Data collection: *APEX2* (Bruker, 2009) ▶; cell refinement: *SAINT* (Bruker, 2009) ▶; data reduction: *SAINT*; program(s) used to solve structure: *SHELXS97* (Sheldrick, 2008 ▶); program(s) used to refine structure: *SHELXL97* (Sheldrick, 2008 ▶); molecular graphics: *ORTEP-3 for Windows* (Farrugia, 2012 ▶) and *PLATON* (Spek, 2009 ▶); software used to prepare material for publication: *WinGX* (Farrugia, 2012 ▶) and *PLATON*.

## Supplementary Material

Crystal structure: contains datablock(s) global, I. DOI: 10.1107/S1600536813021892/hb7118sup1.cif

Structure factors: contains datablock(s) I. DOI: 10.1107/S1600536813021892/hb7118Isup2.hkl

Additional supplementary materials:  crystallographic information; 3D view; checkCIF report

## Figures and Tables

**Table 1:** Hydrogen-bond geometry (Å, °)

*D*—H⋯*A*	*D*—H	H⋯*A*	*D*⋯*A*	*D*—H⋯*A*
N1—H1⋯O1	0.86	1.97	2.616 (4)	131
N1—H1⋯O1^i^	0.86	2.55	3.193 (5)	132

Symmetry code: (i) 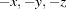.

**Fig. 1.**
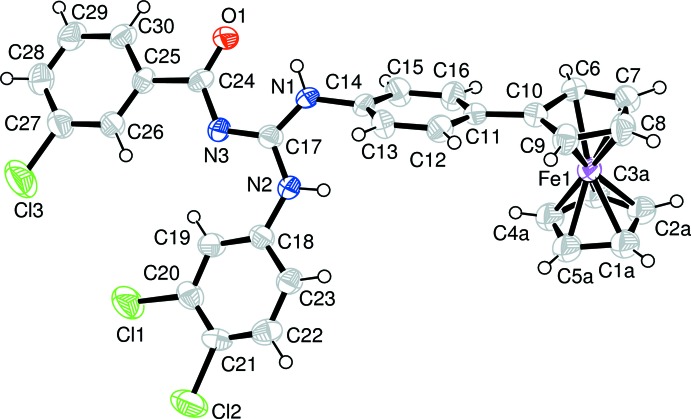
View of the title compound with displacement ellipsoids drawn at the 50% probability level. Only the major part of disordered ferrocene is shown for clarity.

**Fig. 2.**
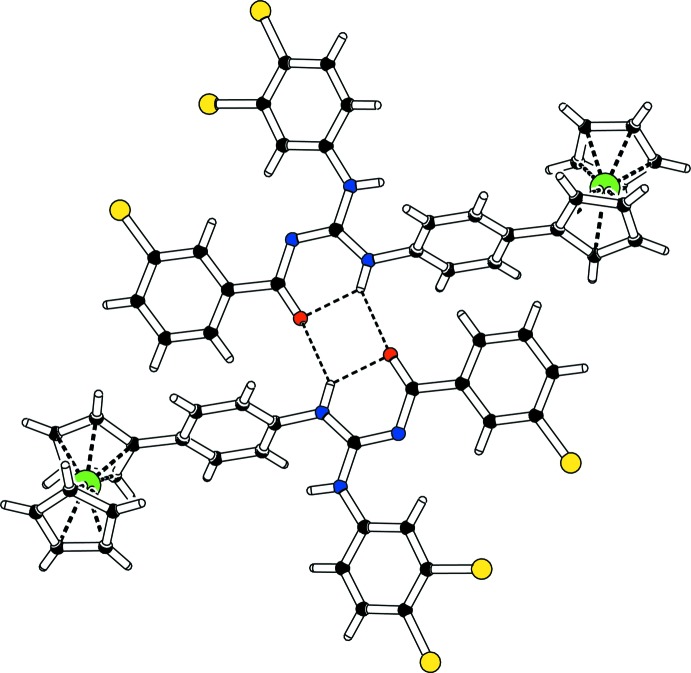
The partial packing, which shows that molecules form dimers.

## supplementary crystallographic information

### 1. Comment

The crystal structure of *p*-ferrocenylaniline (Bequeath *et al.*, 2007) has been published. As part of our studies in this area, the title compound (I, Fig. 1) has been prepared.

In (I), the benzene ring A (C11—C16), dichlorophenyl B (C18—C23/CL1/CL2) and 3-chlorobenzoyl C (O1/C24—C30/CL3) are essentailly planar with r.m.s. deviation of 0.0075, 0.0154 and 0.0512 Å, respectively. The guanidine group D (C17/N1/N2/N3) is also close to planar with r.m.s. deviation of 0.0107 Å from the mean square plane. The dihedral angle between A/B, A/C, A/D, B/C, B/D and C/D is 59.1 (9)°, 52.0 (9)°, 64.8 (2)°, 10.2 (1)°, 16.6 (2)° and 14.8 (2)°, respectively. In the crystal, the molecules are dimerized due to intra and intermolecular H-bondings of N—H···O type (Table 1, Fig. 2).

### 2. Experimental

The synthesis of the compound (I) was achieved in four steps. In the first step; 4- nitrophenylferrocene was made by the coupling of ferrocene with diazonium salts of nitroaniline using phase transfer catalyst (Gul *et al.*, 2013). In the second step; this nitro phenyl ferrocene was reduced into 4-ferrocenylaniline using palladium on charcoal and hydrazine as reducing agent. In the third step, 3-chlorobenzoyl-3,4-dichlorophenyl thiourea was synthesized by the coupling of substituted aniline with thiocynates in acetone. In the fourth step; the thiourea was mixed with the 4-ferrocenyl aniline in dimethylformamide (DMF) in equimolar ratio with two equivalents of triethylamine (Et_3_N). The temperature was maintained below 278 K using an ice bath and one equivalent of mercuric chloride (HgCl_2_) was added to the reaction mixture with vigorous stirring. The ice bath was removed after 30 minutes while the stirring continued overnight. The progress of the reaction was monitored by thin layer chromatography (TLC) till the completion of reaction. Chloroform (CHCl_3_, 20 ml) was added to the reaction mixture and the suspension was filtered through a sintered glass funnel to remove the mercuric sulfide (HgS) residue. The solvents from filtrate were evaporated under reduced pressure and residue was re-dissolved in dichloromethane (CH_2_Cl_2_, 20 ml), washed with water (4 × 30 ml) and dried the organic phase over anhydrous magnesium sulfate (MgSO_4_). The solvent was evaporated and residue was purified by column chromatography to afford orange needles.

### 3. Refinement

The non-coordinating ferrocine ring is disordered over two set of sites with refined occupancy ratio of 0.577 (8):0.423 (8). The disordered rings were treated as regular pentagones and all disordered C-atoms were treated having equal anisotropic displacement parameters.

The H-atoms were positioned geometrically (C–H = 0.93, N—H = 0.86 Å) and refined as riding with *U*_iso_(H) = x*U*_eq_(C, N), where x = 1.2 for all H-atoms.

### Crystal data

**Table:**

[Fe(C_5_H_5_)(C_25_H_17_Cl_3_N_3_O)]	*F*(000) = 1232
*M**_r_* = 602.71	*D*_x_ = 1.548 Mg m^−^^3^
Monoclinic, *P*2_1_/*c*	Mo *K*α radiation, λ = 0.71073 Å
Hall symbol: -P 2ybc	Cell parameters from 2612 reflections
*a* = 17.674 (3) Å	θ = 1.7–25.5°
*b* = 6.1352 (12) Å	µ = 0.92 mm^−^^1^
*c* = 23.961 (5) Å	*T* = 296 K
β = 95.359 (9)°	Needle, orange
*V* = 2586.8 (9) Å^3^	0.32 × 0.15 × 0.14 mm
*Z* = 4	

### Data collection

**Table:**

Bruker Kappa APEXII CCD diffractometer	4828 independent reflections
Radiation source: fine-focus sealed tube	2612 reflections with *I* > 2σ(*I*)
Graphite monochromator	*R*_int_ = 0.088
Detector resolution: 8.00 pixels mm^-1^	θ_max_ = 25.5°, θ_min_ = 1.7°
ω scans	*h* = −21→21
Absorption correction: multi-scan (*SADABS*; Bruker, 2009)	*k* = −7→4
*T*_min_ = 0.757, *T*_max_ = 0.882	*l* = −29→29
20020 measured reflections	

### Refinement

**Table:**

Refinement on *F*^2^	Primary atom site location: structure-invariant direct methods
Least-squares matrix: full	Secondary atom site location: difference Fourier map
*R*[*F*^2^ > 2σ(*F*^2^)] = 0.060	Hydrogen site location: inferred from neighbouring sites
*wR*(*F*^2^) = 0.122	H-atom parameters constrained
*S* = 1.01	*w* = 1/[σ^2^(*F*_o_^2^) + (0.0365*P*)^2^ + 0.9025*P*] where *P* = (*F*_o_^2^ + 2*F*_c_^2^)/3
4828 reflections	(Δ/σ)_max_ < 0.001
319 parameters	Δρ_max_ = 0.45 e Å^−^^3^
0 restraints	Δρ_min_ = −0.36 e Å^−^^3^

### Special details

**Table:**

Geometry. Bond distances, angles *etc*. have been calculated using the rounded fractional coordinates. All su's are estimated from the variances of the (full) variance-covariance matrix. The cell e.s.d.'s are taken into account in the estimation of distances, angles and torsion angles
Refinement. The disordered cyclopentadienyl was refined in two groups as regular pentagons. All the disordered C-atoms were treated anisotropically having equal thermal parameters because refinement anisotropically with individual atoms or rings affoarded large ellipsoids. The sides of regular pentagons after final refinement have naearly 1.392 and 1.436 Å.Refinement of *F*^2^ against ALL reflections. The weighted *R*-factor *wR* and goodness of fit *S* are based on *F*^2^, conventional *R*-factors *R* are based on *F*, with *F* set to zero for negative *F*^2^. The threshold expression of *F*^2^ > σ(*F*^2^) is used only for calculating *R*-factors(gt) *etc*. and is not relevant to the choice of reflections for refinement. *R*-factors based on *F*^2^ are statistically about twice as large as those based on *F*, and *R*-factors based on ALL data will be even larger.

### Fractional atomic coordinates and isotropic or equivalent isotropic displacement parameters (Å^2^)

**Table:**

	*x*	*y*	*z*	*U*_iso_*/*U*_eq_	Occ. (<1)
Fe1	0.41250 (3)	0.69812 (11)	0.12271 (3)	0.0408 (2)	
Cl1	−0.23803 (8)	0.6602 (2)	0.23753 (7)	0.0813 (7)	
Cl2	−0.16675 (8)	1.1155 (2)	0.27386 (7)	0.0793 (7)	
Cl3	−0.37626 (8)	0.2461 (3)	0.13798 (9)	0.1053 (9)	
O1	−0.07255 (16)	−0.0117 (5)	0.03837 (14)	0.0496 (12)	
N1	0.03247 (19)	0.2745 (5)	0.07005 (15)	0.0437 (14)	
N2	−0.0035 (2)	0.5331 (6)	0.13072 (16)	0.0462 (16)	
N3	−0.09308 (18)	0.2824 (6)	0.09663 (15)	0.0370 (12)	
C1A	0.4535 (7)	0.9342 (15)	0.1766 (3)	0.0560 (13)	0.577 (8)
C2A	0.4968 (5)	0.739 (2)	0.1859 (3)	0.0560 (13)	0.577 (8)
C3A	0.4464 (7)	0.5698 (15)	0.1994 (3)	0.0560 (13)	0.577 (8)
C4A	0.3719 (5)	0.6600 (18)	0.1985 (3)	0.0560 (13)	0.577 (8)
C5A	0.3763 (6)	0.8852 (18)	0.1844 (3)	0.0560 (13)	0.577 (8)
C6	0.4013 (3)	0.4461 (8)	0.0682 (2)	0.0492 (19)	
C7	0.4639 (3)	0.5772 (10)	0.0581 (2)	0.059 (2)	
C8	0.4372 (3)	0.7848 (9)	0.0450 (2)	0.0560 (19)	
C9	0.3585 (3)	0.7876 (8)	0.04738 (19)	0.0480 (19)	
C10	0.3351 (2)	0.5776 (7)	0.06239 (19)	0.0403 (17)	
C11	0.2564 (2)	0.5044 (7)	0.06789 (18)	0.0358 (17)	
C12	0.1957 (3)	0.6316 (7)	0.04830 (19)	0.0428 (17)	
C13	0.1216 (2)	0.5604 (7)	0.04920 (19)	0.0431 (17)	
C14	0.1082 (2)	0.3580 (7)	0.07057 (19)	0.0387 (17)	
C15	0.1675 (3)	0.2293 (7)	0.0918 (2)	0.0483 (19)	
C16	0.2414 (3)	0.3030 (8)	0.0902 (2)	0.0495 (19)	
C17	−0.0234 (3)	0.3598 (7)	0.09743 (19)	0.0396 (17)	
C18	−0.0475 (2)	0.6642 (7)	0.16347 (19)	0.0405 (17)	
C19	−0.1152 (3)	0.6010 (7)	0.18288 (19)	0.0455 (17)	
C20	−0.1515 (3)	0.7403 (8)	0.2162 (2)	0.0483 (19)	
C21	−0.1209 (3)	0.9408 (8)	0.2318 (2)	0.0461 (17)	
C22	−0.0541 (3)	1.0020 (8)	0.2122 (2)	0.0486 (19)	
C23	−0.0169 (3)	0.8671 (7)	0.17853 (19)	0.0434 (17)	
C24	−0.1123 (3)	0.0956 (7)	0.06794 (19)	0.0372 (17)	
C25	−0.1913 (2)	0.0199 (7)	0.07554 (18)	0.0368 (17)	
C26	−0.2414 (3)	0.1522 (7)	0.1006 (2)	0.0462 (19)	
C27	−0.3140 (3)	0.0785 (9)	0.1071 (2)	0.056 (2)	
C28	−0.3366 (3)	−0.1253 (9)	0.0883 (2)	0.059 (2)	
C29	−0.2870 (3)	−0.2568 (8)	0.0639 (2)	0.058 (2)	
C30	−0.2147 (3)	−0.1847 (8)	0.05693 (19)	0.0478 (17)	
C1B	0.4078 (9)	0.9420 (19)	0.1807 (5)	0.0560 (13)	0.423 (8)
C2B	0.4799 (7)	0.855 (3)	0.1830 (5)	0.0560 (13)	0.423 (8)
C3B	0.4749 (8)	0.636 (2)	0.1957 (5)	0.0560 (13)	0.423 (8)
C4B	0.3998 (9)	0.589 (2)	0.2011 (5)	0.0560 (13)	0.423 (8)
C5B	0.3583 (7)	0.778 (3)	0.1919 (5)	0.0560 (13)	0.423 (8)
H4A	0.32816	0.58511	0.20578	0.0669*	0.577 (8)
H5A	0.33594	0.98300	0.18091	0.0669*	0.577 (8)
H1	0.02185	0.15928	0.05042	0.0521*	
H1A	0.47233	1.06954	0.16718	0.0669*	0.577 (8)
H2	0.04367	0.56882	0.13204	0.0550*	
H2A	0.54884	0.72513	0.18357	0.0669*	0.577 (8)
H3A	0.45974	0.42573	0.20742	0.0669*	0.577 (8)
H13	0.08132	0.64863	0.03547	0.0515*	
H15	0.15824	0.09371	0.10714	0.0580*	
H16	0.28166	0.21539	0.10445	0.0592*	
H19	−0.13599	0.46521	0.17341	0.0545*	
H22	−0.03344	1.13779	0.22190	0.0583*	
H23	0.02874	0.91081	0.16568	0.0520*	
H26	−0.22644	0.29051	0.11305	0.0557*	
H28	−0.38572	−0.17327	0.09215	0.0702*	
H29	−0.30206	−0.39563	0.05188	0.0696*	
H30	−0.18143	−0.27413	0.03961	0.0571*	
H6	0.40302	0.29859	0.07720	0.0589*	
H7	0.51433	0.53234	0.05996	0.0712*	
H8	0.46676	0.90291	0.03603	0.0669*	
H9	0.32692	0.90767	0.04028	0.0571*	
H12	0.20465	0.76922	0.03406	0.0514*	
H1B	0.39481	1.08645	0.17296	0.0669*	0.423 (8)
H2B	0.52408	0.93006	0.17711	0.0669*	0.423 (8)
H3B	0.51523	0.53839	0.19974	0.0669*	0.423 (8)
H4B	0.38048	0.45272	0.20958	0.0669*	0.423 (8)
H5B	0.30605	0.79144	0.19303	0.0669*	0.423 (8)

### Atomic displacement parameters (Å^2^)

**Table:**

	*U*^11^	*U*^22^	*U*^33^	*U*^12^	*U*^13^	*U*^23^
Fe1	0.0381 (4)	0.0473 (4)	0.0371 (4)	−0.0059 (3)	0.0048 (3)	−0.0050 (3)
Cl1	0.0681 (10)	0.0776 (11)	0.1053 (14)	−0.0083 (8)	0.0463 (9)	−0.0134 (9)
Cl2	0.0849 (11)	0.0645 (11)	0.0927 (13)	0.0146 (8)	0.0305 (9)	−0.0219 (8)
Cl3	0.0615 (10)	0.0872 (13)	0.176 (2)	−0.0093 (8)	0.0578 (11)	−0.0286 (12)
O1	0.044 (2)	0.045 (2)	0.062 (2)	−0.0092 (16)	0.0161 (18)	−0.0213 (17)
N1	0.038 (2)	0.035 (2)	0.059 (3)	−0.0106 (17)	0.010 (2)	−0.0213 (19)
N2	0.037 (2)	0.048 (3)	0.055 (3)	−0.0123 (19)	0.012 (2)	−0.018 (2)
N3	0.035 (2)	0.035 (2)	0.042 (2)	−0.0088 (18)	0.0083 (17)	−0.0039 (19)
C1A	0.057 (2)	0.061 (3)	0.0504 (18)	−0.0074 (19)	0.0073 (19)	−0.0061 (18)
C2A	0.057 (2)	0.061 (3)	0.0504 (18)	−0.0074 (19)	0.0073 (19)	−0.0061 (18)
C3A	0.057 (2)	0.061 (3)	0.0504 (18)	−0.0074 (19)	0.0073 (19)	−0.0061 (18)
C4A	0.057 (2)	0.061 (3)	0.0504 (18)	−0.0074 (19)	0.0073 (19)	−0.0061 (18)
C5A	0.057 (2)	0.061 (3)	0.0504 (18)	−0.0074 (19)	0.0073 (19)	−0.0061 (18)
C6	0.041 (3)	0.048 (3)	0.059 (4)	−0.003 (3)	0.007 (3)	−0.019 (3)
C7	0.039 (3)	0.083 (5)	0.057 (4)	−0.002 (3)	0.009 (3)	−0.023 (3)
C8	0.046 (3)	0.076 (4)	0.047 (3)	−0.017 (3)	0.009 (3)	0.004 (3)
C9	0.044 (3)	0.054 (4)	0.046 (3)	−0.006 (2)	0.005 (2)	0.007 (2)
C10	0.038 (3)	0.043 (3)	0.040 (3)	−0.002 (2)	0.005 (2)	−0.005 (2)
C11	0.036 (3)	0.034 (3)	0.038 (3)	−0.003 (2)	0.007 (2)	−0.006 (2)
C12	0.046 (3)	0.034 (3)	0.049 (3)	−0.004 (2)	0.008 (2)	0.004 (2)
C13	0.035 (3)	0.045 (3)	0.050 (3)	0.001 (2)	0.008 (2)	0.004 (2)
C14	0.037 (3)	0.038 (3)	0.042 (3)	−0.012 (2)	0.008 (2)	−0.010 (2)
C15	0.047 (3)	0.030 (3)	0.070 (4)	−0.003 (2)	0.016 (3)	0.007 (2)
C16	0.041 (3)	0.042 (3)	0.065 (4)	−0.001 (2)	0.002 (2)	0.002 (3)
C17	0.044 (3)	0.035 (3)	0.041 (3)	−0.004 (2)	0.010 (2)	−0.003 (2)
C18	0.040 (3)	0.041 (3)	0.041 (3)	0.004 (2)	0.006 (2)	−0.003 (2)
C19	0.054 (3)	0.039 (3)	0.046 (3)	−0.010 (2)	0.018 (3)	−0.006 (2)
C20	0.047 (3)	0.059 (4)	0.041 (3)	0.002 (3)	0.015 (2)	0.002 (3)
C21	0.059 (3)	0.033 (3)	0.047 (3)	0.009 (2)	0.009 (3)	−0.006 (2)
C22	0.063 (4)	0.038 (3)	0.045 (3)	−0.006 (3)	0.006 (3)	0.000 (2)
C23	0.046 (3)	0.037 (3)	0.048 (3)	−0.005 (2)	0.008 (2)	0.000 (2)
C24	0.047 (3)	0.029 (3)	0.035 (3)	−0.007 (2)	0.000 (2)	0.000 (2)
C25	0.036 (3)	0.036 (3)	0.038 (3)	−0.008 (2)	0.002 (2)	0.006 (2)
C26	0.042 (3)	0.034 (3)	0.064 (4)	−0.006 (2)	0.013 (3)	−0.005 (2)
C27	0.045 (3)	0.056 (4)	0.069 (4)	−0.002 (3)	0.015 (3)	−0.004 (3)
C28	0.047 (3)	0.063 (4)	0.068 (4)	−0.015 (3)	0.014 (3)	0.001 (3)
C29	0.059 (3)	0.050 (4)	0.067 (4)	−0.025 (3)	0.013 (3)	−0.006 (3)
C30	0.053 (3)	0.047 (3)	0.044 (3)	−0.013 (3)	0.008 (2)	−0.007 (3)
C1B	0.057 (2)	0.061 (3)	0.0504 (18)	−0.0074 (19)	0.0073 (19)	−0.0061 (18)
C2B	0.057 (2)	0.061 (3)	0.0504 (18)	−0.0074 (19)	0.0073 (19)	−0.0061 (18)
C3B	0.057 (2)	0.061 (3)	0.0504 (18)	−0.0074 (19)	0.0073 (19)	−0.0061 (18)
C4B	0.057 (2)	0.061 (3)	0.0504 (18)	−0.0074 (19)	0.0073 (19)	−0.0061 (18)
C5B	0.057 (2)	0.061 (3)	0.0504 (18)	−0.0074 (19)	0.0073 (19)	−0.0061 (18)

### Geometric parameters (Å, º)

**Table:**

Fe1—C1A	2.029 (9)	C11—C16	1.382 (7)
Fe1—C2A	2.038 (8)	C12—C13	1.383 (6)
Fe1—C3A	2.037 (8)	C13—C14	1.372 (6)
Fe1—C4A	2.028 (8)	C14—C15	1.372 (6)
Fe1—C5A	2.022 (9)	C15—C16	1.386 (7)
Fe1—C6	2.022 (5)	C18—C19	1.379 (6)
Fe1—C7	2.009 (5)	C18—C23	1.391 (6)
Fe1—C8	2.023 (5)	C19—C20	1.369 (7)
Fe1—C9	2.037 (5)	C20—C21	1.381 (7)
Fe1—C10	2.034 (4)	C21—C22	1.363 (7)
Fe1—C1B	2.049 (12)	C22—C23	1.367 (7)
Fe1—C2B	2.028 (14)	C24—C25	1.499 (6)
Fe1—C3B	2.015 (13)	C25—C26	1.379 (6)
Fe1—C4B	2.026 (12)	C25—C30	1.382 (6)
Fe1—C5B	2.050 (13)	C26—C27	1.383 (7)
Cl1—C20	1.729 (5)	C27—C28	1.376 (8)
Cl2—C21	1.724 (5)	C28—C29	1.363 (7)
Cl3—C27	1.723 (6)	C29—C30	1.377 (7)
O1—C24	1.234 (6)	C1A—H1A	0.9300
N1—C14	1.432 (5)	C1B—H1B	0.9300
N1—C17	1.342 (6)	C2A—H2A	0.9300
N2—C17	1.356 (6)	C2B—H2B	0.9300
N2—C18	1.408 (6)	C3A—H3A	0.9300
N3—C17	1.318 (6)	C3B—H3B	0.9300
N3—C24	1.363 (6)	C4A—H4A	0.9300
N1—H1	0.8600	C4B—H4B	0.9300
N2—H2	0.8600	C5A—H5A	0.9300
C1A—C2A	1.428 (15)	C5B—H5B	0.9300
C1A—C5A	1.426 (16)	C6—H6	0.9300
C1B—C5B	1.38 (2)	C7—H7	0.9300
C1B—C2B	1.38 (2)	C8—H8	0.9300
C2A—C3A	1.425 (15)	C9—H9	0.9300
C2B—C3B	1.38 (2)	C12—H12	0.9300
C3A—C4A	1.427 (15)	C13—H13	0.9300
C3B—C4B	1.38 (2)	C15—H15	0.9300
C4A—C5A	1.426 (15)	C16—H16	0.9300
C4B—C5B	1.38 (2)	C19—H19	0.9300
C6—C7	1.407 (8)	C22—H22	0.9300
C6—C10	1.417 (6)	C23—H23	0.9300
C7—C8	1.384 (8)	C26—H26	0.9300
C8—C9	1.398 (8)	C28—H28	0.9300
C9—C10	1.410 (7)	C29—H29	0.9300
C10—C11	1.479 (5)	C30—H30	0.9300
C11—C12	1.373 (6)		
			
C1A—Fe1—C2A	41.1 (4)	Fe1—C6—C10	70.0 (3)
C1A—Fe1—C3A	69.2 (3)	Fe1—C7—C8	70.5 (3)
C1A—Fe1—C4A	69.4 (4)	Fe1—C7—C6	70.1 (3)
C1A—Fe1—C5A	41.2 (4)	C6—C7—C8	108.0 (5)
C1A—Fe1—C6	164.8 (4)	Fe1—C8—C9	70.4 (3)
C1A—Fe1—C7	126.4 (4)	Fe1—C8—C7	69.4 (3)
C1A—Fe1—C8	107.7 (3)	C7—C8—C9	108.8 (5)
C1A—Fe1—C9	118.8 (3)	Fe1—C9—C10	69.6 (3)
C1A—Fe1—C10	152.7 (3)	C8—C9—C10	108.4 (4)
C2A—Fe1—C3A	40.9 (4)	Fe1—C9—C8	69.3 (3)
C2A—Fe1—C4A	69.2 (3)	Fe1—C10—C11	128.5 (3)
C2A—Fe1—C5A	69.3 (4)	C9—C10—C11	126.9 (4)
C2A—Fe1—C6	126.5 (4)	C6—C10—C9	106.6 (4)
C2A—Fe1—C7	105.4 (3)	C6—C10—C11	126.4 (4)
C2A—Fe1—C8	116.5 (3)	Fe1—C10—C9	69.8 (3)
C2A—Fe1—C9	151.0 (3)	Fe1—C10—C6	69.1 (3)
C2A—Fe1—C10	165.7 (4)	C10—C11—C12	120.5 (4)
C3A—Fe1—C4A	41.1 (4)	C10—C11—C16	121.5 (4)
C3A—Fe1—C5A	69.3 (4)	C12—C11—C16	117.9 (4)
C3A—Fe1—C6	106.9 (3)	C11—C12—C13	121.7 (4)
C3A—Fe1—C7	115.9 (3)	C12—C13—C14	119.2 (4)
C3A—Fe1—C8	149.5 (4)	N1—C14—C13	120.9 (3)
C3A—Fe1—C9	167.8 (4)	N1—C14—C15	118.6 (4)
C3A—Fe1—C10	128.5 (3)	C13—C14—C15	120.5 (4)
C4A—Fe1—C5A	41.2 (4)	C14—C15—C16	119.4 (4)
C4A—Fe1—C6	118.0 (3)	C11—C16—C15	121.2 (4)
C4A—Fe1—C7	150.6 (4)	N1—C17—N3	125.6 (4)
C4A—Fe1—C8	168.4 (3)	N1—C17—N2	115.6 (4)
C4A—Fe1—C9	130.5 (3)	N2—C17—N3	118.8 (4)
C4A—Fe1—C10	108.9 (3)	N2—C18—C19	124.7 (4)
C5A—Fe1—C6	152.6 (3)	N2—C18—C23	115.7 (4)
C5A—Fe1—C7	165.9 (3)	C19—C18—C23	119.5 (4)
C5A—Fe1—C8	129.3 (3)	C18—C19—C20	119.3 (4)
C5A—Fe1—C9	109.9 (3)	Cl1—C20—C21	120.5 (4)
C5A—Fe1—C10	119.2 (3)	Cl1—C20—C19	118.1 (4)
C6—Fe1—C7	40.9 (2)	C19—C20—C21	121.4 (5)
C6—Fe1—C8	67.9 (2)	Cl2—C21—C20	121.2 (4)
C6—Fe1—C9	67.9 (2)	Cl2—C21—C22	120.0 (4)
C6—Fe1—C10	40.91 (19)	C20—C21—C22	118.8 (5)
C1B—Fe1—C6	171.5 (5)	C21—C22—C23	121.1 (5)
C2B—Fe1—C6	147.0 (5)	C18—C23—C22	119.9 (5)
C3B—Fe1—C6	115.4 (4)	N3—C24—C25	113.0 (4)
C4B—Fe1—C6	109.5 (4)	O1—C24—C25	119.4 (4)
C5B—Fe1—C6	132.9 (5)	O1—C24—N3	127.6 (5)
C7—Fe1—C8	40.2 (2)	C24—C25—C30	119.8 (4)
C7—Fe1—C9	68.0 (2)	C26—C25—C30	119.3 (4)
C7—Fe1—C10	68.94 (19)	C24—C25—C26	120.9 (4)
C1B—Fe1—C7	147.4 (4)	C25—C26—C27	119.8 (4)
C2B—Fe1—C7	116.5 (4)	Cl3—C27—C26	119.3 (4)
C3B—Fe1—C7	110.4 (4)	Cl3—C27—C28	120.4 (4)
C4B—Fe1—C7	133.6 (4)	C26—C27—C28	120.3 (5)
C5B—Fe1—C7	172.2 (5)	C27—C28—C29	120.0 (5)
C8—Fe1—C9	40.3 (2)	C28—C29—C30	120.2 (5)
C8—Fe1—C10	68.30 (19)	C25—C30—C29	120.4 (4)
C1B—Fe1—C8	117.3 (4)	Fe1—C1A—H1A	127.00
C2B—Fe1—C8	111.6 (4)	C2A—C1A—H1A	126.00
C3B—Fe1—C8	134.5 (4)	C5A—C1A—H1A	126.00
C4B—Fe1—C8	172.9 (5)	Fe1—C1B—H1B	126.00
C5B—Fe1—C8	147.0 (5)	C2B—C1B—H1B	126.00
C9—Fe1—C10	40.53 (19)	C5B—C1B—H1B	126.00
C1B—Fe1—C9	111.0 (4)	C3A—C2A—H2A	126.00
C2B—Fe1—C9	134.3 (5)	Fe1—C2A—H2A	127.00
C3B—Fe1—C9	173.4 (4)	C1A—C2A—H2A	126.00
C4B—Fe1—C9	145.8 (5)	C1B—C2B—H2B	126.00
C5B—Fe1—C9	115.9 (4)	Fe1—C2B—H2B	125.00
C1B—Fe1—C10	132.9 (4)	C3B—C2B—H2B	126.00
C2B—Fe1—C10	172.0 (5)	C4A—C3A—H3A	126.00
C3B—Fe1—C10	145.6 (4)	Fe1—C3A—H3A	127.00
C4B—Fe1—C10	114.4 (4)	C2A—C3A—H3A	126.00
C5B—Fe1—C10	109.1 (4)	C2B—C3B—H3B	126.00
C1B—Fe1—C2B	39.5 (6)	Fe1—C3B—H3B	124.00
C1B—Fe1—C3B	66.6 (5)	C4B—C3B—H3B	126.00
C1B—Fe1—C4B	66.3 (5)	Fe1—C4A—H4A	127.00
C1B—Fe1—C5B	39.2 (6)	C5A—C4A—H4A	126.00
C2B—Fe1—C3B	40.0 (6)	C3A—C4A—H4A	126.00
C2B—Fe1—C4B	66.7 (6)	C3B—C4B—H4B	126.00
C2B—Fe1—C5B	66.3 (5)	C5B—C4B—H4B	126.00
C3B—Fe1—C4B	39.8 (6)	Fe1—C4B—H4B	125.00
C3B—Fe1—C5B	66.6 (5)	Fe1—C5A—H5A	126.00
C4B—Fe1—C5B	39.5 (6)	C4A—C5A—H5A	126.00
C14—N1—C17	125.9 (4)	C1A—C5A—H5A	126.00
C17—N2—C18	130.5 (4)	C1B—C5B—H5B	126.00
C17—N3—C24	119.9 (4)	C4B—C5B—H5B	126.00
C14—N1—H1	117.00	Fe1—C5B—H5B	126.00
C17—N1—H1	117.00	Fe1—C6—H6	127.00
C17—N2—H2	115.00	C7—C6—H6	126.00
C18—N2—H2	115.00	C10—C6—H6	126.00
Fe1—C1A—C2A	69.8 (5)	C8—C7—H7	126.00
Fe1—C1A—C5A	69.1 (5)	Fe1—C7—H7	125.00
C2A—C1A—C5A	108.0 (9)	C6—C7—H7	126.00
Fe1—C1B—C5B	70.4 (8)	C9—C8—H8	126.00
C2B—C1B—C5B	108.1 (13)	Fe1—C8—H8	126.00
Fe1—C1B—C2B	69.4 (8)	C7—C8—H8	126.00
Fe1—C2A—C3A	69.5 (5)	Fe1—C9—H9	127.00
C1A—C2A—C3A	108.0 (9)	C8—C9—H9	126.00
Fe1—C2A—C1A	69.1 (5)	C10—C9—H9	126.00
C1B—C2B—C3B	107.9 (12)	C11—C12—H12	119.00
Fe1—C2B—C1B	71.1 (7)	C13—C12—H12	119.00
Fe1—C2B—C3B	69.5 (8)	C14—C13—H13	120.00
Fe1—C3A—C4A	69.1 (5)	C12—C13—H13	120.00
C2A—C3A—C4A	108.0 (9)	C16—C15—H15	120.00
Fe1—C3A—C2A	69.6 (4)	C14—C15—H15	120.00
Fe1—C3B—C4B	70.5 (7)	C11—C16—H16	119.00
Fe1—C3B—C2B	70.5 (7)	C15—C16—H16	119.00
C2B—C3B—C4B	107.8 (12)	C18—C19—H19	120.00
C3A—C4A—C5A	108.0 (8)	C20—C19—H19	120.00
Fe1—C4A—C3A	69.8 (4)	C21—C22—H22	120.00
Fe1—C4A—C5A	69.2 (5)	C23—C22—H22	119.00
Fe1—C4B—C5B	71.2 (7)	C18—C23—H23	120.00
Fe1—C4B—C3B	69.7 (7)	C22—C23—H23	120.00
C3B—C4B—C5B	108.2 (12)	C25—C26—H26	120.00
Fe1—C5A—C1A	69.6 (5)	C27—C26—H26	120.00
C1A—C5A—C4A	108.0 (9)	C27—C28—H28	120.00
Fe1—C5A—C4A	69.6 (5)	C29—C28—H28	120.00
C1B—C5B—C4B	107.9 (12)	C28—C29—H29	120.00
Fe1—C5B—C4B	69.3 (8)	C30—C29—H29	120.00
Fe1—C5B—C1B	70.3 (7)	C25—C30—H30	120.00
Fe1—C6—C7	69.1 (3)	C29—C30—H30	120.00
C7—C6—C10	108.2 (4)		
			
C2A—Fe1—C1A—C5A	−119.5 (7)	C5A—Fe1—C9—C10	−112.0 (4)
C3A—Fe1—C1A—C2A	37.5 (6)	C6—Fe1—C9—C8	−81.3 (3)
C3A—Fe1—C1A—C5A	−82.0 (6)	C6—Fe1—C9—C10	38.7 (3)
C4A—Fe1—C1A—C2A	81.6 (5)	C7—Fe1—C9—C8	−37.1 (3)
C4A—Fe1—C1A—C5A	−37.9 (5)	C7—Fe1—C9—C10	82.9 (3)
C5A—Fe1—C1A—C2A	119.5 (7)	C8—Fe1—C9—C10	120.0 (4)
C7—Fe1—C1A—C2A	−69.9 (6)	C10—Fe1—C9—C8	−120.0 (4)
C7—Fe1—C1A—C5A	170.6 (5)	C1A—Fe1—C10—C6	167.4 (6)
C8—Fe1—C1A—C2A	−110.3 (5)	C1A—Fe1—C10—C9	49.6 (7)
C8—Fe1—C1A—C5A	130.3 (5)	C1A—Fe1—C10—C11	−72.2 (8)
C9—Fe1—C1A—C2A	−152.7 (4)	C3A—Fe1—C10—C6	−69.4 (5)
C9—Fe1—C1A—C5A	87.9 (5)	C3A—Fe1—C10—C9	172.8 (5)
C10—Fe1—C1A—C2A	173.0 (5)	C3A—Fe1—C10—C11	51.0 (6)
C10—Fe1—C1A—C5A	53.5 (8)	C4A—Fe1—C10—C6	−111.2 (4)
C1A—Fe1—C2A—C3A	119.8 (8)	C4A—Fe1—C10—C9	131.0 (4)
C3A—Fe1—C2A—C1A	−119.8 (8)	C4A—Fe1—C10—C11	9.2 (5)
C4A—Fe1—C2A—C1A	−82.1 (6)	C5A—Fe1—C10—C6	−155.2 (4)
C4A—Fe1—C2A—C3A	37.6 (6)	C5A—Fe1—C10—C9	87.0 (4)
C5A—Fe1—C2A—C1A	−37.9 (6)	C5A—Fe1—C10—C11	−34.8 (5)
C5A—Fe1—C2A—C3A	81.9 (6)	C6—Fe1—C10—C9	−117.8 (4)
C6—Fe1—C2A—C1A	167.9 (5)	C6—Fe1—C10—C11	120.5 (5)
C6—Fe1—C2A—C3A	−72.4 (6)	C7—Fe1—C10—C6	37.5 (3)
C7—Fe1—C2A—C1A	128.4 (5)	C7—Fe1—C10—C9	−80.3 (3)
C7—Fe1—C2A—C3A	−111.9 (6)	C7—Fe1—C10—C11	158.0 (4)
C8—Fe1—C2A—C1A	86.8 (6)	C8—Fe1—C10—C6	80.8 (3)
C8—Fe1—C2A—C3A	−153.4 (5)	C8—Fe1—C10—C9	−37.0 (3)
C9—Fe1—C2A—C1A	56.1 (8)	C8—Fe1—C10—C11	−158.8 (4)
C9—Fe1—C2A—C3A	175.9 (6)	C9—Fe1—C10—C6	117.8 (4)
C1A—Fe1—C3A—C2A	−37.6 (6)	C9—Fe1—C10—C11	−121.7 (5)
C1A—Fe1—C3A—C4A	82.1 (6)	C17—N1—C14—C13	−63.4 (6)
C2A—Fe1—C3A—C4A	119.7 (8)	C17—N1—C14—C15	118.1 (5)
C4A—Fe1—C3A—C2A	−119.7 (8)	C14—N1—C17—N2	−3.9 (6)
C5A—Fe1—C3A—C2A	−81.9 (6)	C14—N1—C17—N3	179.8 (4)
C5A—Fe1—C3A—C4A	37.9 (6)	C18—N2—C17—N1	177.8 (4)
C6—Fe1—C3A—C2A	126.8 (5)	C18—N2—C17—N3	−5.7 (7)
C6—Fe1—C3A—C4A	−113.5 (5)	C17—N2—C18—C19	22.5 (7)
C7—Fe1—C3A—C2A	83.8 (6)	C17—N2—C18—C23	−160.5 (4)
C7—Fe1—C3A—C4A	−156.5 (5)	C24—N3—C17—N1	2.7 (7)
C8—Fe1—C3A—C2A	52.0 (8)	C24—N3—C17—N2	−173.5 (4)
C8—Fe1—C3A—C4A	171.7 (6)	C17—N3—C24—O1	−4.5 (7)
C10—Fe1—C3A—C2A	166.6 (5)	C17—N3—C24—C25	174.9 (4)
C10—Fe1—C3A—C4A	−73.6 (6)	Fe1—C1A—C2A—C3A	−58.8 (5)
C1A—Fe1—C4A—C3A	−81.6 (6)	C5A—C1A—C2A—Fe1	58.8 (5)
C1A—Fe1—C4A—C5A	37.9 (6)	C5A—C1A—C2A—C3A	0.0 (8)
C2A—Fe1—C4A—C3A	−37.5 (6)	Fe1—C1A—C5A—C4A	59.2 (5)
C2A—Fe1—C4A—C5A	82.0 (6)	C2A—C1A—C5A—Fe1	−59.2 (5)
C3A—Fe1—C4A—C5A	119.5 (8)	C2A—C1A—C5A—C4A	0.0 (8)
C5A—Fe1—C4A—C3A	−119.5 (8)	Fe1—C2A—C3A—C4A	−58.6 (5)
C6—Fe1—C4A—C3A	83.7 (6)	C1A—C2A—C3A—Fe1	58.5 (5)
C6—Fe1—C4A—C5A	−156.8 (5)	C1A—C2A—C3A—C4A	0.0 (8)
C7—Fe1—C4A—C3A	47.0 (9)	Fe1—C3A—C4A—C5A	−58.8 (5)
C7—Fe1—C4A—C5A	166.5 (6)	C2A—C3A—C4A—Fe1	58.9 (5)
C9—Fe1—C4A—C3A	167.6 (5)	C2A—C3A—C4A—C5A	0.0 (8)
C9—Fe1—C4A—C5A	−72.9 (6)	Fe1—C4A—C5A—C1A	−59.2 (5)
C10—Fe1—C4A—C3A	127.5 (5)	C3A—C4A—C5A—Fe1	59.2 (5)
C10—Fe1—C4A—C5A	−113.1 (5)	C3A—C4A—C5A—C1A	0.0 (8)
C1A—Fe1—C5A—C4A	−119.3 (7)	Fe1—C6—C7—C8	−60.6 (4)
C2A—Fe1—C5A—C1A	37.7 (5)	C10—C6—C7—Fe1	59.3 (3)
C2A—Fe1—C5A—C4A	−81.6 (6)	C10—C6—C7—C8	−1.3 (6)
C3A—Fe1—C5A—C1A	81.6 (6)	Fe1—C6—C10—C9	60.1 (3)
C3A—Fe1—C5A—C4A	−37.7 (6)	Fe1—C6—C10—C11	−123.1 (5)
C4A—Fe1—C5A—C1A	119.3 (7)	C7—C6—C10—Fe1	−58.7 (3)
C6—Fe1—C5A—C1A	168.4 (5)	C7—C6—C10—C9	1.4 (5)
C6—Fe1—C5A—C4A	49.1 (9)	C7—C6—C10—C11	178.2 (4)
C8—Fe1—C5A—C1A	−70.1 (6)	Fe1—C7—C8—C9	−59.6 (3)
C8—Fe1—C5A—C4A	170.6 (4)	C6—C7—C8—Fe1	60.3 (3)
C9—Fe1—C5A—C1A	−111.3 (5)	C6—C7—C8—C9	0.8 (6)
C9—Fe1—C5A—C4A	129.3 (5)	Fe1—C8—C9—C10	−58.8 (3)
C10—Fe1—C5A—C1A	−155.0 (4)	C7—C8—C9—Fe1	58.9 (3)
C10—Fe1—C5A—C4A	85.7 (5)	C7—C8—C9—C10	0.1 (6)
C2A—Fe1—C6—C7	−69.6 (5)	Fe1—C9—C10—C6	−59.6 (3)
C2A—Fe1—C6—C10	170.7 (4)	Fe1—C9—C10—C11	123.6 (5)
C3A—Fe1—C6—C7	−110.3 (5)	C8—C9—C10—Fe1	58.6 (3)
C3A—Fe1—C6—C10	130.0 (4)	C8—C9—C10—C6	−0.9 (5)
C4A—Fe1—C6—C7	−153.3 (4)	C8—C9—C10—C11	−177.7 (4)
C4A—Fe1—C6—C10	87.0 (4)	Fe1—C10—C11—C12	104.8 (5)
C5A—Fe1—C6—C7	172.3 (6)	Fe1—C10—C11—C16	−78.4 (5)
C5A—Fe1—C6—C10	52.6 (7)	C6—C10—C11—C12	−164.1 (5)
C7—Fe1—C6—C10	−119.7 (4)	C6—C10—C11—C16	12.7 (7)
C8—Fe1—C6—C7	37.8 (3)	C9—C10—C11—C12	12.1 (7)
C8—Fe1—C6—C10	−82.0 (3)	C9—C10—C11—C16	−171.1 (5)
C9—Fe1—C6—C7	81.4 (3)	C10—C11—C12—C13	175.2 (4)
C9—Fe1—C6—C10	−38.3 (3)	C16—C11—C12—C13	−1.7 (7)
C10—Fe1—C6—C7	119.7 (4)	C10—C11—C16—C15	−175.6 (4)
C1A—Fe1—C7—C6	168.4 (4)	C12—C11—C16—C15	1.3 (7)
C1A—Fe1—C7—C8	−73.1 (5)	C11—C12—C13—C14	0.5 (7)
C2A—Fe1—C7—C6	128.6 (4)	C12—C13—C14—N1	−177.3 (4)
C2A—Fe1—C7—C8	−113.0 (4)	C12—C13—C14—C15	1.3 (7)
C3A—Fe1—C7—C6	86.1 (4)	N1—C14—C15—C16	176.9 (4)
C3A—Fe1—C7—C8	−155.5 (4)	C13—C14—C15—C16	−1.7 (7)
C4A—Fe1—C7—C6	53.8 (7)	C14—C15—C16—C11	0.4 (7)
C4A—Fe1—C7—C8	172.2 (6)	N2—C18—C19—C20	177.6 (4)
C6—Fe1—C7—C8	118.5 (4)	C23—C18—C19—C20	0.7 (7)
C8—Fe1—C7—C6	−118.5 (4)	N2—C18—C23—C22	−177.4 (4)
C9—Fe1—C7—C6	−81.3 (3)	C19—C18—C23—C22	−0.2 (7)
C9—Fe1—C7—C8	37.2 (3)	C18—C19—C20—Cl1	177.5 (4)
C10—Fe1—C7—C6	−37.6 (3)	C18—C19—C20—C21	−1.5 (7)
C10—Fe1—C7—C8	80.9 (3)	Cl1—C20—C21—Cl2	1.1 (6)
C1A—Fe1—C8—C7	126.1 (4)	Cl1—C20—C21—C22	−177.3 (4)
C1A—Fe1—C8—C9	−114.0 (4)	C19—C20—C21—Cl2	−179.9 (4)
C2A—Fe1—C8—C7	82.6 (5)	C19—C20—C21—C22	1.7 (7)
C2A—Fe1—C8—C9	−157.5 (4)	Cl2—C21—C22—C23	−179.6 (4)
C3A—Fe1—C8—C7	47.4 (7)	C20—C21—C22—C23	−1.2 (7)
C3A—Fe1—C8—C9	167.3 (6)	C21—C22—C23—C18	0.4 (7)
C5A—Fe1—C8—C7	166.7 (5)	O1—C24—C25—C26	−169.5 (4)
C5A—Fe1—C8—C9	−73.4 (5)	O1—C24—C25—C30	10.1 (6)
C6—Fe1—C8—C7	−38.4 (3)	N3—C24—C25—C26	11.1 (6)
C6—Fe1—C8—C9	81.5 (3)	N3—C24—C25—C30	−169.3 (4)
C7—Fe1—C8—C9	119.9 (4)	C24—C25—C26—C27	180.0 (4)
C9—Fe1—C8—C7	−119.9 (4)	C30—C25—C26—C27	0.4 (7)
C10—Fe1—C8—C7	−82.7 (3)	C24—C25—C30—C29	179.7 (4)
C10—Fe1—C8—C9	37.3 (3)	C26—C25—C30—C29	−0.7 (7)
C1A—Fe1—C9—C8	83.5 (5)	C25—C26—C27—Cl3	−179.5 (4)
C1A—Fe1—C9—C10	−156.5 (4)	C25—C26—C27—C28	−0.5 (7)
C2A—Fe1—C9—C8	45.0 (7)	Cl3—C27—C28—C29	180.0 (4)
C2A—Fe1—C9—C10	165.0 (6)	C26—C27—C28—C29	1.0 (7)
C4A—Fe1—C9—C8	170.1 (5)	C27—C28—C29—C30	−1.3 (7)
C4A—Fe1—C9—C10	−69.9 (5)	C28—C29—C30—C25	1.2 (7)
C5A—Fe1—C9—C8	128.0 (4)		

### Hydrogen-bond geometry (Å, º)

**Table:**

*D*—H···*A*	*D*—H	H···*A*	*D*···*A*	*D*—H···*A*
N1—H1···O1	0.86	1.97	2.616 (4)	131
N1—H1···O1^i^	0.86	2.55	3.193 (5)	132

Symmetry code: (i) −*x*, −*y*, −*z*.
